# Functional connectivity alterations between default mode network and occipital cortex in patients with obsessive-compulsive disorder (OCD)

**DOI:** 10.1192/j.eurpsy.2021.1955

**Published:** 2021-08-13

**Authors:** T. Geffen, J. Smallwood, C. Finke, Z. Sjoerds, F. Schlagenhauf

**Affiliations:** 1 Psychiatry, Charité, Berlin, Germany; 2 Psyhcology, Queen’s University, Kingston, Canada; 3 Neurology, Charité, Berlin, Germany; 4 Berlin School Of Mind And Brain, Humboldt-Universität, Berlin, Germany; 5 Institute Of Psychology - Cognitive Psychology Unit, Leiden University, Leiden, Netherlands; 6 Leiden Institute For Brain & Cognition, Leiden University, Leiden, Netherlands; 7 Institute For Human Cognitive And Brain Sciences, Max-Planck, Leipzig, Germany; 8 Bernstein Center For Computational Neuroscience, Humboldt-Universität, Berlin, Germany

**Keywords:** neuropsychiatry, functional-connectivity, DMN_Visual-Network, ocd

## Abstract

**Introduction:**

A meta-analysis by Gürsel et al. (2018) found altered functional connectivity in OCD patients within and between default mode (DMN), salience (SN), and frontoparietal networks (FPN), as well as evidence for aberrant fronto-striatal circuitry.

**Objectives:**

Testing the replicability of meta-analysis rsfMRI findings in OCD patients.

**Methods:**

We measured functional connectivity during resting-state fMRI in a sample of OCD patients (n=24) and controls matched for age and sex (n=33). The CONN toolbox implemented in SPM was used to perform seed-to-voxel analysis using 30 seed regions based on the previous meta-analytic findings.

**Results:**

OCD patients showed reduced functional connectivity between SN and DMN compared to controls, replicating previous findings. We did not observe significant group differences of functional connectivity within the DMN, SN, or FPN. The strongest finding consisted of altered connectivity between DMN and SN to the visual network. OCD patients showed reduced functional connectivity between the left lateral parietal seed (LPl) and the inferior lateral occipital pole left (iLOCl) compared to controls. Furthermore, the LPl was found to be hyperconnected with the right superior lateral occipital cortex (sLOCr) and the right precuneus. This finding was positively correlated to OCD symptom severity, especially compulsions.

**Conclusions:**

Our findings replicated partly the meta-analysis findings, specifically reduced connectivity between SN and DMN. Using seeds based on the meta-analysis, we identified aberrations between the SN and, in particular, the DMN to the visual network. This raises the question about the visual system’s involvement in OCD symptoms and the abnormal connectivity of a unimodal region to the multimodal DMN.

**Disclosure:**

No significant relationships.

